# Barriers to uptake of cervical cancer screening services in low-and-middle-income countries: a systematic review

**DOI:** 10.1186/s12905-022-02043-y

**Published:** 2022-12-02

**Authors:** Z. Petersen, A. Jaca, T. G. Ginindza, G. Maseko, S. Takatshana, P. Ndlovu, N. Zondi, N. Zungu, C. Varghese, G. Hunting, G. Parham, P. Simelela, S. Moyo

**Affiliations:** 1 Human & Social Capabilities (HSC), Human Sciences Research Council, Pretoria, South Africa; 2 Cochrane South Africa, South African Medical Research Council, Cape Town, South Africa; 3 Public Health Medicine, University of KwaZulu-Natal (UKZN), Durban, South Africa; 4 Cancer & Infectious Diseases Epidemiology Research Unit (CIDERU), Durban, South Africa; 5 Cervical Cancer Elimination Initiative, World Health Organization, Geneva, Switzerland; 6 School of Public Health and Family Medicine, University of Cape Town, Cape Town, South Africa

**Keywords:** Cervical cancer, Screening, Social ecological model, Low-and-middle-income countries

## Abstract

**Objectives:**

Low-and-middle-income countries (LMICs) bear a disproportionate burden of cervical cancer mortality. We aimed to identify what is currently known about barriers to cervical cancer screening among women in LMICs and propose remedial actions.

**Design:**

This was a systematic review using Medical Subject Headings (MeSH) terms in Google Scholar, PubMed, Scopus, and Web of Science databases. We also contacted medical associations and universities for grey literature and checked reference lists of eligible articles for relevant literature published in English between 2010 and 2020. We summarized the findings using a descriptive narrative based on themes identified as levels of the social ecological model.

**Setting:**

We included studies conducted in LMICs published in English between 2010 and 2020.

**Participants:**

We included studies that reported on barriers to cervical cancer screening among women 15 years and older, eligible for cervical cancer screening.

**Results:**

Seventy-nine articles met the inclusion criteria. We identified individual, cultural/traditional and religious, societal, health system, and structural barriers to screening. Lack of knowledge and awareness of cervical cancer in general and of screening were the most frequent individual level barriers. Cultural/traditional and religious barriers included prohibition of screening and unsupportive partners and families, while social barriers were largely driven by community misconceptions. Health system barriers included policy and programmatic factors, and structural barriers were related to geography, education and cost. Underlying reasons for these barriers included limited information about cervical cancer and screening as a preventive strategy, poorly resourced health systems that lacked policies or implemented them poorly, generalised limited access to health services, and gender norms that deprioritize the health needs of women.

**Conclusion:**

A wide range of barriers to screening were identified across most LMICs. Urgent implementation of clear policies supported by health system capacity for implementation, community wide advocacy and information dissemination, strengthening of policies that support women’s health and gender equality, and targeted further research are needed to effectively address the inequitable burden of cervical cancer in LMICs.

**Supplementary Information:**

The online version contains supplementary material available at 10.1186/s12905-022-02043-y.

## Key messages

What is already known: Low-and-middle-income countries (LMICs) bear a disproportionate burden of cervical cancer mortality and there is limited knowledge on barriers to cervical cancer screening uptake across LMICS.

Findings: Women in LMICs face individual level, cultural/traditional and religious, societal, health system, and structural barriers to cervical cancer screening. The underlying reasons for these barriers include limited information about cervical cancer and screening as a preventive strategy, poorly resourced health systems without screening policies, poorly implemented policies, generalised limited access to health services, and gender norms that deprioritize the health needs of women.

What the findings imply: There is a need for education, information dissemination, and advocacy to dispel myths about cervical cancer, and implementation of clear cervical cancer policies and guidelines with prerequisite structures and resources across diverse health settings. Policies that support sexual and reproductive health and the rights of women should be strengthened and expanded and account for inequities in access for diverse groups of women. Education and awareness initiatives should be driven by local and community contexts, and engage community members and multiple stakeholders, including traditional and religious figures. In addition, the introduction and roll out of more modern screening approaches in LMICs should be prioritized to ensure more women are reached.

## Introduction

Cervical cancer, although preventable and curable, is the fourth most common cancer among women globally [1]. The burden is greatest in low-and-middle-income countries (LMICs) with age-standardized incidence rates varying from 75/100000 women in highest-risk countries to less than 10/100000 women in lowest risk countries [1]. In 2018, approximately 90% of deaths occurred in LMICs [2]. The remarkable geographic contrasts in cervical cancer incidence and mortality reflect differences in social and structural contexts associated with cervical cancer, and inequities in access to information about cervical cancer, prevention, screening, and effective cancer treatment facilities and thus indicate areas with the greatest need for interventions [3]. Consequently, the World Health Organization’s (WHO) global strategy to accelerate the elimination of cervical cancer proposes a vision of a world where cervical cancer is eliminated as a public health problem by employing measures that are sensitive to women’s needs, their social circumstances, and the personal, cultural, social, structural and economic barriers hindering their access to health services [2].

With almost all cervical cancer cases (99%) linked to human papillomaviruses infection (HPV), HPV vaccination is a key primary preventive strategy, with secondary prevention – screening - remaining a key component of the cervical cancer elimination toolkit, especially where there is low HPV vaccination availability, access, and uptake [3, 4]. Screening coverage of eligible women in most LMICs is on average 19%, compared to 63% in high income countries, and thus it is important to review identified barriers to screening uptake to address the burden in LMICs [4].

We conducted a systematic review on barriers to uptake of cervical cancer screening services (including poor provision of services) in LMICs. The objectives of the review were to i) document and investigate the underlying reasons for poor uptake of cervical cancer screening services in LMICs, ii) identify research gaps, and iii) provide evidence for decision-making and policy interventions for improved programmes and actions to support the elimination of cervical cancer in LMICs. We used Brofenbrenner’s social ecological model [5, 6] to understand the dynamic interrelations among personal and environmental factors. First introduced in the 1970s as a conceptual model, the social ecological model was formalized as a theory in the 1980s and underwent revisions by Bronfenbrenner until his death in 2005. In his initial theory, Bronfenbrenner proposed that to understand human development, the entire ecological system in which growth occurs needs to be considered. In subsequent revisions, the model examines how human beings develop according to their environment, which includes society and the context which impacts behavior and development.

## Methods

The review was conducted according to the Preferred Reporting Items for Systematic Reviews and Meta-Analyses (PRISMA) guidelines and included LMICs, as defined by the World Bank based on per capita gross national income in 2020 [7]. The research question was framed using the broad population, concept and context (PCC) framework recommended by the Joanna Briggs Institute for Scoping Reviews [8] and was defined as: “What are the barriers to the uptake of cervical cancer screening services in LMICs?”. The population was women (15 years and older) eligible for cervical cancer screening. Studies that examined HPV vaccination and included girls younger than 15 years old together with older girls and women were also included.

### Search strategy

Two authors (AJ and ZP) developed the search strategy. A comprehensive literature search was conducted in February 2021 in Scopus, Web of Science and Pubmed. No language or date restrictions were applied in the initial search. A search in Google Scholar using the keywords ‘cervical cancer screening’ and ‘barriers to cervical cancer screening’ was also conducted, aimed at finding studies that may not have been included in the findings from the major databases that were searched. We also searched the websites of the WHO, the International Agency for Research on Cancer (IARC), and the reference lists of all included studies for additional relevant articles. The search was initiated with keywords and refined by adapting search terms from relevant literature to include a variation of the terminology used in different countries. The detailed search strategy for the three databases is shown in Table 1.

**Table 1:** Search strategies

Search #	Search Texts and Syntaxes	Date	Output
PubMed			
#1	“Uterine Cervical Neoplasms” OR “cervical neoplasm” OR “Cervical cancer” OR “cervix neoplasm” OR “cervix cancer” AND	22/02/2021	
#2	“Vaginal Smears” OR Papanicolaou OR “pap smear” OR “pap stain” OR “pap test” OR “vaginal smear” OR “Mass Screening” OR “Early Diagnosis” OR “cervical screening” OR “Cervical cancer examination” OR “early detection” OR “early diagnosis” OR early detect* AND		
#3	barrie* OR obstacle* OR challeng* AND		
#4	Afghanistan* OR Albania OR Algeria OR Angola OR Argentina OR Armenia OR Azerbaijan OR Bangladesh OR Belarus OR Beliz OR Benin OR Bhutan OR Bolivia OR Bosnia OR Herzegovin OR Botswana OR Brazil OR Bulgaria OR “Burkina Faso” OR Burundi OR “Cabo Verde” OR Cambodia OR Cameroon OR “Central Africa” OR Chad OR China OR Chinese OR Colombia OR “Comoro Islands’ OR Congo OR “Costa Rica” OR “Cote d’Ivoir” OR “Ivory Coast” OR Cuba OR Djibouti OR “Dominican Republic” OR Ecuador OR Egypt OR “El Salvador” OR Eritrea OR Ethiopia OR Fiji OR Gabon OR Gambia OR Ghana OR “Guinea Bissau” OR Kenya* OR Lesotho* OR Liberia* OR Libya* OR Macedonia* OR Madagascar OR Malawi OR Malaysia OR Mali OR * OR Mauritius OR Morocco* OR Mozambique OR Namibia OR Niger OR Nigeria OR Pakistan OR Rwanda OR “Sao Tome” OR Senegal OR Seychelles OR “Sierra Leon” OR Somalia OR South Africa OR Sudan OR “Sri Lanka” OR Tanzania OR Togo OR Tunisia OR Uganda OR Zambia OR Zimbabwe OR Africa* OR resource-poor OR low-resource OR limited-resource OR resource-constrain* OR under-resource* OR poor*-resource* OR resource-scarce* OR scarce*-resource* OR low-income OR middle-income OR “low income” OR “middle income” or LMIC*		
#1 AND #2 AND #3 AND #4			385 articles
Scopus			
#1	*“Uterine Cervical Neoplasms”* OR *“cervical neoplasm”* OR *“Cervical cancer”* OR *“cervix neoplasm”* OR *“cervix cancer”* AND *“Vaginal Smears”* OR *papanicolaou* OR *“pap smear”* OR *“pap stain”* OR *“pap test”* OR *“vaginal smear”* OR *“Mass Screening”* OR *“Early Diagnosis”* OR *“cervical screening”* OR *“Cervical cancer examination”* OR *“early detection”* OR *“early diagnosis”* OR *early* AND *detect* AND *barrier* OR *obstacle* OR *challenge* AND *afghanistan* OR *albania* OR *algeria* OR *angola* OR *argentina* OR *armenia* OR *azerbaijan* OR *bangladesh* OR *belarus* OR *beliz* OR *benin* OR *bhutan* OR *bolivia* OR *bosnia* OR *herzegovin* OR *botswana* OR *brazil* OR *bulgaria* OR *“Burkina Faso”* OR *burundi* OR *“Cabo Verde”* OR *cambodia* OR *cameroon* OR *“Central Africa”* OR *chad* OR *china* OR *chinese* OR *colombia* OR *“Comoro Islands”* OR *congo* OR *“Costa Rica”* OR *“Cote d’Ivoir”* OR *“Ivory Coast”* OR *Cuba* OR *djibouti* OR *“Dominican Republic”* OR *ecuador* OR *egypt* OR *“El Salvador”* OR *eritrea* OR *ethiopia* OR *fiji* OR *gabon* OR *gambia* OR *ghana* OR *“Guinea Bissau”* OR *kenya* OR *lesotho* OR *liberia* OR *libya* OR *macedonia* OR *madagascar* OR *malawi* OR *malaysia* OR *mali* OR *mauritius* OR *morocco* OR *mozambique* OR *namibia* OR *niger* OR *nigeria* OR *pakistan* OR *rwanda* OR *“Sao Tome”* OR *senegal* OR *seychelles* OR *“Sierra Leon”* OR *somalia* OR *“South Africa”* OR *sudan* OR *“Sri Lanka”* OR *tanzania* OR *togo* OR *tunisia* OR *Uganda* OR *zambia* OR *zimbabwe* OR *africa* OR *resource-poor* OR *low-resource* OR *limited-resource* OR *resource-constrain* OR *under-resource* OR *poor-resource* OR *resource-scarce* OR *scarce-resource* OR *low-income* OR *middle-income* OR *“low income”* OR *“middle income”* OR *lmic*	24/02/2021	1280 articles
Web of Science			
#1	*TS = “Uterine Cervical Neoplasms”* OR *TS = “cervical neoplasm”* OR *TS = “Cervical cancer”* OR *TS = “cervix neoplasm”* OR *TS = “cervix cancer”* AND *TS = “Vaginal Smears”* OR *TS = papanicolaou* OR *TS = “pap smear” TS=*OR *“pap stain”* OR *TS = “pap test”* OR *TS = “vaginal smear”* OR *TS = “Mass Screening”* OR *TS = “Early Diagnosis”* OR *TS = “cervical screening”* OR *TS = “Cervical cancer examination”* OR *TS = “early detection”* OR *TS = “early diagnosis”* OR *TS = early OR TS = detect* AND *TS = barrier* OR *TS = obstacle* OR *challenge* AND *TS = afghanistan* OR *TS = albania* OR *TS = algeria* OR *TS = angola* OR *TS = argentina* OR *TS = armenia* OR *TS = azerbaijan* OR *TS = bangladesh* OR *TS = belarus* OR *TS = beliz* OR *TS = benin* OR *TS = bhutan* OR *TS = bolivia* OR *TS = bosnia* OR *TS = herzegovin* OR *TS = botswana* OR *TS = brazil* OR *TS = bulgaria* OR *TS = “Burkina Faso”* OR *TS = burundi* OR *TS = “Cabo Verde”* OR *TS = cambodia* OR *TS = cameroon* OR *TS = “Central Africa”* OR *TS = chad* OR *TS = china* OR *TS = chinese* OR *TS = colombia* OR *TS = “Comoro Islands”* OR *TS = congo* OR *TS = “Costa Rica”* OR *TS = “Cote d’Ivoir”* OR *TS = “Ivory Coast”* OR *TS = cuba* OR *TS = djibouti* OR *TS = “Dominican Republic”* OR *TS = ecuador* OR *TS = egypt* OR *TS = “El Salvador”* OR *TS = eritrea* OR *TS = ethiopia* OR *TS = fiji* OR *TS = gabon* OR *TS = gambia* OR *TS = ghana* OR *TS = “Guinea Bissau”* OR *TS = kenya* OR *TS = lesotho* OR *TS = liberia* OR *TS = libya* OR *TS = macedonia* OR *TS = madagascar* OR *TS = malawi* OR *TS = malaysia* OR *TS = mali* OR *TS = mauritius* OR *TS = morocco* OR *TS = mozambique* OR *TS = namibia* OR *TS = niger* OR *TS = nigeria* OR *TS = pakistan* OR *TS = rwanda* OR *TS = “Sao Tome”* OR *TS = senegal* OR *TS = seychelles* OR *TS = “Sierra Leon”* OR *TS = somalia* OR *TS = “South Africa”* OR *TS = sudan* OR *TS = “Sri Lanka”* OR *TS = tanzania* OR *TS = togo* OR *TS = tunisia* OR *TS=Uganda* OR *TS = zambia* OR *TS = zimbabwe* OR *TS = africa* OR *TS = resource-poor* OR *TS = low-resource* OR *TS = limited-resource* OR *TS = resource-constrain* OR *TS = under-resource* OR *TS = poor-resource* OR *TS = resource-scarce* OR *TS = scarce-resource* OR *TS = low-income* OR *TS = middle-income* OR *TS = “low income”* OR *TS = “middle income”* OR *TS = lmic*	26/02/2021	461 articles
	*Google scholar*		18
	*Google and networks*		4
Total number of articles retrieved			2148 articles

Studies addressing barriers to and uptake of cervical cancer screening in LMICs and published in English over 10 years (1 January 2010 to December 2020) were eligible for inclusion. Project and academic reports including Master’s and Doctoral theses were also eligible while editorials, commentaries, and abstracts where we could not access full-text articles were ineligible. Working in pairs, the authors independently screened the titles and abstracts of the search output and retrieved the full texts of those considered eligible. The authors then independently assessed the full texts for inclusion and resolved disagreements through discussion and consensus.

### Data extraction

A standardized data extraction tool was used. Information was extracted on the country of study, aim/s, design, population, sample size, participant ages, screening type, documented barriers, reported findings, and recommendations. Discrepancies were resolved through discussion and consensus. Two authors assessed the quality of the studies included using the Critical Appraisal Skill Program(CASP) tool [8]. See Appendix 1, Quality Assessment of studies.

## Results

### Search Results

**Fig. 1:**
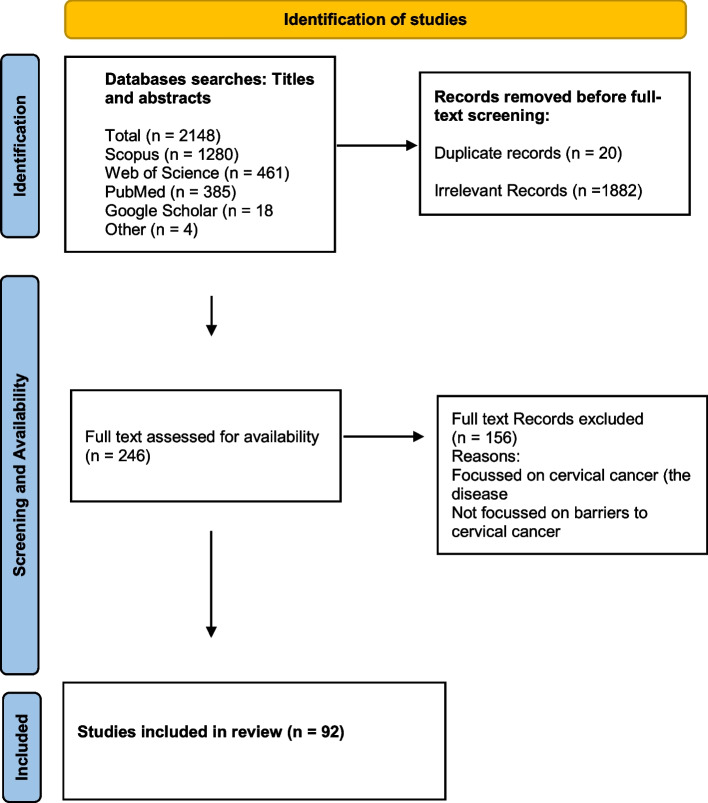
Search strategy flow diagram

The literature search yielded a total of 2148 articles: 385 from PubMed, 1280 from Scopus, and 461 from Web of Science, 18 from Google and Google scholar. After removing 20 duplicates, we screened titles for eligibility and 1882 irrelevant articles were excluded (Fig. 1). Full texts of the 246 remaining articles were assessed for eligibility, and 92 met the inclusion criteria. Thirteen review articles were excluded, leaving 79 articles based on individual studies.

### Characteristics of included studies

The included studies were undertaken in 28 LMICs; with 61% undertaken in Africa, 21% in Asia, 5% in North America, 9% in South America, 1% in Oceania and 3% in Europe. The characteristics of the included studies are shown in Table 2. Of the included individual studies, 45 (57%) were quantitative, 27 (34%) qualitative and 4 (5%) used a combination of qualitative and quantitative methods. Four studies were based on secondary data analysis [9–12]. The quantitative studies were largely cross-sectional surveys, while the qualitative studies involved focus group discussions, in-depth and semi-structured interviews (Table 2).

**Table 2:** Studies reporting barriers to Cervical cancer screening in low- and middle-income countries

*Author, Country, Year*	*Study design*	*Population*	*Sample size*	*Age (years)*	*Screening method*	*Barriers*	*Quality rating*
**Africa**							
Getahun T et al. Ethiopia, 2020 [13]	Mixed methods Cross-sectional and IDIs	Rural and urban women, Cervical cancer screening service providers	821, 10 in-depth interviews	median age 39 years (range 30–49)	Not specified	Individual, Social, Health system	High
Megersa BS et al. Ethiopia, 2020 [14]	Qualitative IDIs and FGDs	Women who had participated in HPV self-sampling, sample collectors, community health care workers	47 (25 -FGDs, 22-in IDIs).	mean age 36 years	HPV self-sampling	Individual, Social, Cultural/ traditional/ religious	High
Ampofo AG et al. Ghana, 2020 [15]	Quantitative Cross-sectional survey	Women of reproductive age	200	15–50	VIA, pap smear	Individual, Social, Cultural/traditional/religious, Health system	High
Calys-Tagoe BNL et al. Ghana, 2020 [9]	Quantitative Secondary data analysis	Women	2711	Mainly ≥50 years	Pap smear	Individual, Cultural/traditional/religious, Structural	Medium
Stewart K et al. Nigeria, 2020 [10]	Quantitative Secondary data analysis	Women who had received cervical screening	621	Not specified	VIA/VILI, and pap smear	Structural	High
Harries J et al. South Africa, 2020 [16]	Qualitative semi-structured interviews	Women with potential breast or Cervical cancer symptoms in urban and rural areas	18	mean age 34.5 years (range 22–58)	Not specified	Individual, Social, Health system, Structural	High
Nyamambi E et al. Zimbabwe, 2020 [17]	Quantitative Cross-sectional	Sexually active women	156	15–50	VIA	Individual, Cultural/traditional/ religious, Health system, Structural	High
Getachew et al. Ethiopia, 2019 [18]	Mixed methods Cross sectional and FGDs	Women of reproductive age attending primary health centres	520	mean age 27.7 years (range 20–49)	Not specified	Individual, Health system, Structural	High
Nigussie T et al. Ethiopia, 2019 [19]	Quantitative Cross sectional	Women from a rural community	737	mean age 36.6 years (range 30–49)	VIA	Individual, Health system	High
Solomon K et al. Ethiopia, 2019 [20]	Quantitative Cross sectional	HIV positive women attending ARV clinics	475	18 + years	VIA	Individual, Cultural/traditional/ religious	Medium
Williams MS et al. Ghana, 2019 [21]	Quantitative Cross-sectional	Women	288	mean age 32.4 years (range 19–64)	Pap smear	Individual, Cultural/traditional/ religious	High
Adewumi K et al. Kenya, 2019 [22]	Quantitative Cross-sectional	Women, community health volunteers	604	not specified	self-collected vaginal swabs for HPV	Social, Cultural/ traditional/religious, Structural	High
Oketch SY et al. Kenya, 2019 [23]	Qualitative IDIs	Women in Cervical cancer screening campaign	120	mean age 36.1 years	HPV self-sampling	Individual, Social, Structural	High
Lieber M et al. South Africa, 2019 [24]	Mixed method IDIs, FGDs observations, chart reviews	Women patients and healthcare providers	12 patients, 3 healthcare providers	Not specified	VIA	Individual, Health system	Medium
Shiferaw S et al. Ethiopia, 2018 [25]	Mixed method, Cross-sectional and qualitative IDIs	HIV-positive women attending heath facilities	581	mean age 35 years (range 21–65)	Not specified	Individual, Cultural/traditional/ religious, Health system, Structural	High
Kangmennaang J et al. Kenya, 2018 [26]	Quantitative Cross-sectional survey, secondary analysis of survey data	Women of reproductive age	14,741	mean age 30 years (range 15–49)	Not specified	Individual, Social, Cultural/ traditional/religious, Structural	High
Ng’ang’a A et al. Kenya, 2018 [27]	Quantitative Nested case-control study in a cross-sectional survey	Women	1180	30–49	Not specified	Individual, Structural	High
Maree JE & Kampinda-Banda M. Malawi, 2020 [28]	Quantitative Cross-sectional	Women, convenient sample in rural district	282	mean age 36.1 years (range 30–45)	VIA	Individual, Health system	High
Keneema M et al. Uganda, 2018 [29]	Quantitative Cross-sectional	Women attending antenatal clinic	100	25–49	not specified	Individual, Cultural/traditional/ religious, Health system, Structural	Medium
Vhuromu EN et al. South Africa, 2018 [30]	Quantitative Cross-sectional	Women attending health clinics	500	20–59	pap-smear	Individual, Cultural/ traditional/religious, Health system	Medium
Kokuro Ml. Ghana, 2017 Thesis [31]	Quantitative Cross-sectional	Women attending reproductive health services	369	18 years+	Not specified	Individual, Social, Cultural/traditional/religious, Structural	High
Bishwajit G & Kpoghomou M, Kenya, 2017 [32]	Quantitative Cross-sectional secondary data analysis	Women	11,138	mean age 29.6 years range (15–49)	Not specified	Structural	High
Lunsford NB et al. Kenya, 2017 [33]	Qualitative FGDs	Women, married men with partners 25–49	100 (10 focus groups)	Women 25–49, men ≥18	Pap versus VIA/VILI	Individual, Social, Cultural/Traditional/religious, Health systems, Structural	High
Tiruneh FN et al. Kenya, 2017 [34]	Quantitative Cross-sectional	Married women	6498	15–49	Pap- smear, urine sampling	Social, Structural	High
Filade TE et al. Nigeria, 2017 [35]	Qualitative FGDs and IDIs	Pregnant women in antenatal care, Healthcare workers	82 pregnant women, 13 Healthcare workers	pregnant women, mean age 28.9 years	HPV DNA based tests	Individual, Cultural/traditional/religious, Health system, Structural	High
Momberg M. et al. South Africa, 2017 [36]	Qualitative FGDs	Women, first time colposcopy clinic attendees	27	mean age 34 years (range 18–49)	pap smear & colposcopy	Individual, Social	High
Malambo N. & Erikson S. Swaziland, 2018 [37]	Qualitative	Women, healthcare workers	20 women, 7 healthcare workers	19–49	Not specified	Individual, Health system	Medium
Mitchell SM et al. Uganda, 2017 [38]	Quantitative Cross-sectional	HIV+ women attending a routine care	87	30–69	Not specified	Individual, Health system	High
Koneru A et al. Tanzania, 2017 [39]	Quantitative Cross-sectional	HIV+ women	399	19 years+	VIA and colposcopy	Individual, Health system	High
Modibbo IF et al. Nigeria, 2016 [40]	Qualitative FGDs	Muslim and Christian women purposively sampled	49	18 years+ mean age 33 years	Not specified	Individual, Social, Cultural/traditional/ religious, Health systems	High
Hweissa NAb et al. Libya, 2016 [41]	Qualitative IDIs	Healthcare workers from public and private sectors	16	Not specified	Pap smear	Individual, Cultural/traditional/religious, Health system, Structural	High
Adepoju EG et al. Nigeria, 2016 [42]	Quantitative Cross-sectional	Women	287	age 51.6 years (SD 14.3)	Pap smear, colposcopy	Individual	Low
Ndejjo R et al. Uganda, 2016 [43]	Quantitative Cross sectional	Women from predominantly rural districts	900	25–49	Not specified	Individual, Social, Health systems, Structural	High
Hasahya OT et al. Uganda, 2016 [44]	Qualitative FGDs	Women of whose daughters had received HPV vaccination.	36	25–49	Not specified	Individual, Social, Health system, Structural	High
Ghidei et al. Ethiopia and Tanzania, 2015 Research report [45]	Descriptive Cross sectional	Women	23	19–45	VIA	Individual, Cultural/traditional/ religious	Low
Compaore SC. et al. Burkina Faso, 2015 [46]	Quantitative Cross-sectional	Women	351	Not specified	VIA, VILI	Individual, Structural	Medium
Munthali CM. et al. Malawi, 2015 [47]	Qualitative interviews	Healthcare workers, cervical screening service providers, District coordinators	53	Not specified	VIA	Individual, Cultural/traditional/religious, Health system, Structural	High
Learmonth D. et al. South Africa, 2015 [48]	Qualitative FGDs	Women of low socioeconomic status	15	25–51	Not specified	Individual, Social, Cultural/traditional/ religious, Health system, Structural	High
Ebu NI et al. Ghana, 2014 [49]	Quantitative Cross-sectional	Women	392	10–74	Pap smear	Individual, Social, Cultural/traditional/ religious, Health system, Structural	High
Omondi Aduda DS & Mkhize N. Kenya, 2014 [50]	Qualitative FGDs	Women screened for syphilis and Cervical cancer	Not specified	not specified	Not specified	Individual, Health system, Structural	High
Kibicho et al. Kenya, 2014 Thesis [51]	Quantitative Cross -sectional	Women of reproductive age in a gynaecology ward	138	mean age 31.6 years (Range 18–49)	Pap smear, coloscopy, VIA/VILI test	Individual, Cultural/traditional/religious, Health system, Structural	High
Abdulkadir IR. Ethiopia, 2013 Thesis [52]	Quantitative cross-sectional	Female university students	392	Mean age 23.3 years (range 18–52	Pap smear	Individual, Cultural/traditional/religious, Health system, Structural	High
Atuhaire L. Uganda, 2013 Thesis [53]	Qualitative Exploratory and descriptive	Women accessing maternal and child health services	25	18–64	all screening	Individual, Health system	High
Mwaka AD et al. Uganda, 2013 [54]	Qualitative	Healthcare workers	15	Not specified	Not specified	Individual, Cultural/traditional/religious, Health system, Structural	High
Paul et al. Peru, Uganda, Vietnam, 2013 [55]	Qualitative	Women, Healthcare workers, village health team	109	not specified	VIA	Individual, Social, Health system, Structural	High
Ngugi et al. Kenya, 2012 [56]	Qualitative IDIs	Women	50	Not specified	Not specified	Individual, Social, Cultural/traditional/religious, Health system, Structural	High
Hyacinth et al. Nigeria, 2012 [57]	Quantitative Cross sectional	Women in their workplace	388	18–65	Pap smear	Individual, Health system	High
Mupepi SC et al. Zimbabwe, 2011 [58]	Quantitative cross-sectional	Sexually active women		12–84	Pap smear	Individual, Social, Cultural/traditional/religious, Health system, Structural	High
**Asia**							
Andersen JG et al. Nepal, 2020 [59]	Qualitative FGDs and IDIs	Women, female community health volunteers	48	30–60	Not specified	Individual, Social, Cultural/traditional, Health system, Structural	High
Spagnoletti BRM et al. Indonesia, 2019 [60]	Qualitative FGDs and semi-structured interviews	Married women and men	56 women, 30 men	women 22–57, men 35–45	VIA and pap smear	Individual, Social, Health system, Structural	High
Gu et al. China, 2018 [61]	Qualitative Semi-structured interviews	Women at risk for cervical cancer in a prior study	27	25–50	pap smear test	Individual, Health system, Structural	High
Ashtarian H et al. Iran, 2017 [62]	Quantitative Cross-sectional	Women attending health centres	355	mean age 34.08 years	pap-smear	Individual, Health system	High
Osth J. Sri Lanka, 2015 Thesis [63]	Quantitative Cross-sectional	Male and female undergraduate students	326	18–30	Pap smear, cytological screening	Individual	High
Jia Y. et al. China, 2013 [64]	Quantitative Cross-sectional	Women	5929	25–65	Pap smear, VIA, colposcopy	Individual, Cultural/traditional/religious	High
Baskaran P. et al. Malaysia, 2013 [65]	Quantitative Cross-sectional	Women attending outpatient care	369	mean age 37.5 years (range 21–65)	Pap smear	Individual, Cultural/traditional/religious, Health system	High
Gan et al. Malaysia, 2013 [66]	Quantitative Cross-sectional	women from 1000 households	959	mean age 45.2 years (range 20–64)	Pap smear	Individual, Social	High
Demirtas B & Acikgoz I. Turkey, 2013 [67]	Quantitative Cross-sectional	women registering at a gynaecology outpatient clinic	256	21–62	Pap Smear	Individual	High
Guvenc et al. Turkey, 2013 [68]	Quasi-experimental	Women	294	21+	Pap smear	Individual	
Reis et al. Turkey, 2012 [69]	Qualitative	Women at gynaecology and obstetrics outpatient clinics	387	Not specified	Pap Smear	Individual	Medium
Gu et al. China, 2012 [70]	Quantitative Cross-sectional	Women	167	25–50	Not specified	Individual	High
Abdullah et al. Malaysia, 2011 [71]	Quantitative Cross-sectional	Female secondary school teachers	403	not specified	Pap smear	Individual, Health system	Medium
Gu et al. China, 2010 [72]	Quantitative Cross-sectional	Women	184	25–50	Not specified	Individual, Health system	Medium
Abdullah & Su, Malaysia, 2010 [73]	Qualitative Semi-structured interviews	Policy makers, healthcare workers	11	37–57	Pap smear	Individual, Health system	
Al-Naggar RA, Isa ZM. Malaysia, 2010 [74]	Quantitative Cross-sectional survey	female Malaysian university students	287	18 years+	Pap smear	Individual, Cultural/traditional/religious, Health system	Medium
Park SJ, & Park Wl. Korea, 2010 [11]	Quantitative Secondary analysis	Women aged 21+, no hysterectomy, eligible for Pap smears	2590	21 years+	Pap smear	Individual, Structural	High
**North America**							
Gottschlich A et al. Guatemala, 2020 [12]	Quantitative Secondary data analysis	screened and unscreened women	15,317	25–49	Pap smear	Individual, Social, Structural	High
Bien-Aimé et al. Haiti, 2020 Thesis [75]	Quantitative Cross sectional	women in five urban areas	200	25 years+	Pap smear, Colposcopy, VIA, HPV test	Individual, Social	Medium
Lyons KD et al. Honduras, 2020 [76]	Quantitative Cross sectional	Rural women	874	Not specified	HPV PCR and pap smear	Individual, Structural	Medium
Chary AN & Rohloff PJ. Guatemala, 2014 [77]	Qualitative Semi-structured interviews	NGO service provider staff	36	Not specified	VIA	Health system	
**South America**							
Barret BW et al. Peru, 2020 [78]	Quantitative Cross sectional	Rural women	619	18–65	HPV testing, VIA or pap smear	Structural	Medium
Collins JH et al. Peru 2019 [79]	Quantitative Cross sectional	Rural women	121 women	mean age 42 years (range 21–76)	not specified	Individual, Structural	High
Nugus P et al. Ecuador, 2018 [80]	Qualitative FGDs and semi structured interviews	Women who had participated in a community-based Cervical cancer screening program	28	24–69	Pap smear	Individual, Social, Cultural/traditional/religious, Health system, Structural	High
Albuquerque et al. Brazil, 2014 [81]	Quantitative Cross sectional	Rural and urban women	493	Mean age 35.4 years (range15–69)	Pap smear	Individual, Health system, Structural	High
Stormo et al. Bolivia, 2012 [82]	Qualitative Descriptive survey	Healthcare workers	42	Not specified	VIA, cryotherapy	Individual, Social, Cultural/traditional/religious, Health system	Medium
Paz-Soldán VA, et al. Peru,2012 [83]	Qualitative semi-structured interviews	Policy makers and healthcare workers	30	Not specified	Not specified	Health system, Structural	High
Paolino M & Arrossi Sl. Argentina, 2011 [84]	Quantitative	Women attending hospital	200	18+	Pap smear	Individual, Health system, Structural	High
**Oceania**							
Townsend JS et al. US Affiliated Pacific Island Jurisdictions (USAPIJ), 2014 [85]	Quantitative cross-sectional	Healthcare workers	72	not specified	HPV testing, Pap smear	Health system, Structural	High
**Europe**							
Valerianova Z. et al. Bulgaria, 2010 [86]	Qualitative	Healthcare workers		23–65	Not specified	Individual, Health system	Medium
Rada C. et al. Romania, 2010 [87]	Quantitative Cross sectional	Men, women	1902	15–82	Pap smear	Individual, Social	Low

*FGDs* Focus group discussions, *IDI* In-depth interviews, VIA Visual inspection with acetic acid, *VILI* Visual inspection with Lugol’s iodine, *HPV* Human papillomavirus

### Patient and Public Involvement

Patients were not directly involved or recruited into this study. We reviewed published articles that investigated the barriers to cervical cancer screening uptake by women in LMICs. The results will be disseminated through a publicly available research report and a manuscript and in conferences and webinars. They will also be distributed through the WHO and the institutions involved in the project.

### Participants

The individual studies included participants from rural and urban areas, women living with and without HIV, women in the general public, women attending antenatal services, university students, and healthcare workers. Four studies included men [33, 60, 63, 87] and in two of the studies, they were partners of women participants [33, 63] while in the others they were university male students. Thirteen studies included healthcare workers exclusively or with non-healthcare workers [13, 14, 24, 35, 37, 41, 47, 54, 55, 73, 82, 85, 86]. Eight studies included participants younger than 18 years old including one study that included girls from the age of 10 years together with older women [15, 17, 26, 32, 34, 49, 58, 87] -36. In 17 studies, age details were not specified (Table 2). Frequently missing information was age of the participants, type of screening and when the study was conducted. The sample sizes of studies ranged from 15 participants [24, 48, 54] to 15,317 participants in a study that analysed secondary data [26].

### Types of screening methods

Forty eight percent of studies were about Papanicolaou (pap) smears exclusively or in combination with other screening methods, 25% about visual inspection with acetic acid (VIA) or visual inspection with Lugol’s iodine (VILI), 5% on HPV screening (through self-sampling or using DNA based tests) exclusively or in combination with other screening methods, while a total of 30% of studies did not specify the type of screening method (Table 2).

### Analysis

Since most studies identified were descriptive or qualitative in design, we analysed and summarized the main findings using a descriptive narrative, based on themes identified as levels of the social ecological model [88]. During the thematic analysis six authors in groups of two grouped the barriers that were identified into five categories, as defined below.

- Individual/personal level barriers – obstacles experienced at individual level
- Cultural/traditional and religious barriers – cultural, traditional, and religious views, norms, and expectations
- Social barriers – community and societal obstacles
- Health system barriers – factors in the design, function and implementation of health systems that make it difficult for some individuals to access, use or benefit from care
- Structural barriers– macroscale obstacles that affected some women disproportionately

**Fig. 2:**
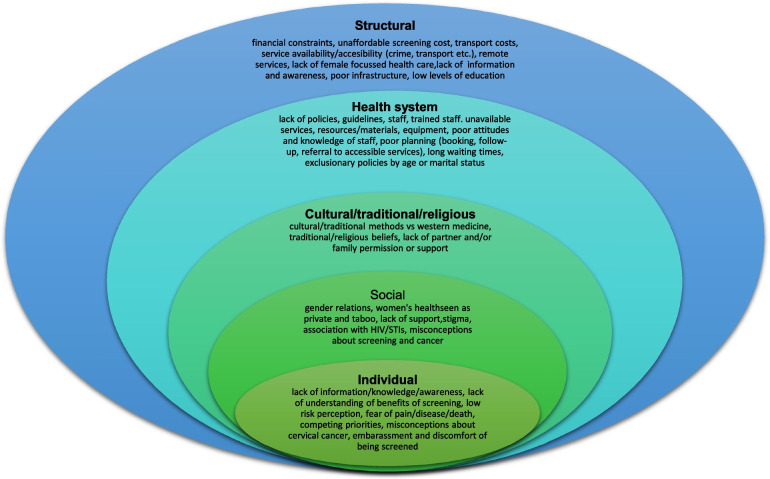
Examples of barriers in the five categories

These categories are not entirely distinct or mutually exclusive as factors in one category overlap and are influenced by those in other categories (Refer to Fig. 2 for a visual diagram depicting barriers across each level).

The barriers to uptake of cervical cancer are interconnected and operate across and within the various levels of the social ecological model. The following category levels include factors that contribute to barriers to cervical cancer screening, spanning the patient/individual level to the structural level. The studies reviewed included quantitative and qualitative input from both women and men (including patients, women from the community, male and female students, female teachers and male partners), as well as from the health service-level (including nurses, doctors, community health workers, policy makers, NGO staff and district coordinators). Information about the different categories of barriers that were identified across the articles included in this review are provided in Table 3.

**Table 3:** Description of the different categories of barriers to cervical cancer screening in low-to-middle-income countries

*Number of articles by continent N = 79*		*Barrier categories*				
Continents	*n*	Individual	Social	Cultural/traditional/religious	Health system	Structural
Africa	*48*	44 (55.6%)	19 (24%)	26 (32.9%)	32 (40.5%)	30 (37.9%)
Asia	*17*	16 (20.2%)	3 (3.7%)	4 (5%)	9 (11.3%)	3 (3.7%)
North America	*4*	3 (3.7%)	2 (2.5%)	–	1(1.2%)	2 (2.5%)
South America	*7*	5 (6.3%)	2 (2.5%)	2 (2.5%)	5 (6.3%)	6 (7.5%)
Oceania	*1*	–	–	–	1(1.2%)	1 (1.2%)
Europe	*2*	2 (2.5%)	1 (1.2%)	–	1(1.2%)	–
Total	*79*	70^a^ (88.6%)	27^a^ (34.1%)	32^a^ (40.5%)	49^a^ (62%)	42^a^ (53.1%)

^a^Total number of articles that described Individual, Social, Cultural/traditional/religious, Health system and Structural barriers

### Individual/personal level barriers

Most studies reported individual or personal level barriers to screening. The most common individual level barriers were lack of knowledge and information about cervical cancer and cervical cancer screening, and its benefits, including women who did not understand the value of screening – i. e., health examination in the absence of symptoms or ill health [18–21, 25, 31, 43, 47–49, 51, 52, 56, 58, 60, 64, 76, 81]. Another commonly reported individual level barrier was fear of receiving positive screening results with many women believing that a cancer diagnosis was terminal [15, 19–21, 25, 31, 33, 35–37, 40, 44, 46, 48–50, 52, 53, 60, 62, 64, 69, 74].

Studies also reported that women had misconceptions about screening and the screening process. Women feared pain from the screening procedure and had misconceptions about possible harms such as contracting cancer, or damage to the uterus or cervix during screening [13–15, 17, 23, 30, 39, 44, 47–49, 51, 54, 56, 61, 62, 65, 81]. In Nigeria, women reported being afraid of contracting infections from the screening equipment or from other sources within the health facility [35, 40]. In Ethiopia, most women offered self-sampling for HPV thought that the process would be painful, while some feared using the Evalyn brush [14], and in South Africa, some women reported fear of concurrent HIV testing during screening for cervical cancer [16].

In 33% of studies conducted in Africa, Asia, and South America many women reported being embarrassed to be screened or to undergo pelvic examination [13, 15, 18, 19, 21, 23, 29–31, 33, 39, 44, 47, 49, 57, 60–67, 69, 71, 72, 81]. Embarrassment was associated with the activity of going to a facility for screening, the pelvic examination itself, and being examined by a male or young healthcare worker [60, 61, 65].

Studies also reported that women, regardless of geography or employment status, faced competing priorities and responsibilities and thus often had limited time to attend screening [28, 73].

### Cultural/traditional /religious and social barriers

Cultural/religious/traditional, and social barriers were closely intertwined in the studies evaluated. Eleven studies reported that women were not screened because of religious or traditional reasons and prohibitions [14, 15, 17, 22, 25, 26, 33, 35, 49, 52, 72]. Two studies reported on possible clashes between western and traditional views of cervical cancer screening [48, 80], and mistrust of western medicine and preference for traditional medicine was reported in Ghana, and South Africa [15, 48]. In Ecuador, there were competing interpretations of health between healthcare workers and the community [80]. Some studies (21%) also reported that men disapproved of cervical cancer screening, with some refusing for their wives to be screened [17, 18, 25, 27, 29, 30, 33, 38, 43, 49, 53, 54, 57, 58, 67, 74, 76]. Other studies reported that women’s health issues, including sexual and reproductive health, were deprioritized and not awarded the same urgency as other health issues [12, 82], while others reported that cervical cancer screening was viewed as a private and taboo topic (culturally embarrassing) not to be discussed, due to its connection to sexual and reproductive health [82].

Social barriers were related to community disapproval or negative community perceptions about the health system, the screening process, lack of peer support, and stigmatization of cervical cancer and the screening process [13, 14, 22, 27, 36, 40, 44, 55, 59, 60, 82]. In some studies stigma was related to cervical cancer being viewed as a terminal disease by some [15, 23], while in others stigma was due to association with sexual transmission, with women attending screening sometimes assumed to be engaged in infidelity or promiscuity [22, 33]. In South Africa where concurrent HV testing was offered, stigma was related to the association of cervical cancer with HIV infection [36].

### Health system barriers

Heath system barriers included lack of capacity, poor organization of services, lack of knowledge about cervical cancer amongst healthcare workers, lack of promotion of screening, poor (negative and unfriendly) attitudes of healthcare workers when interacting with patients, and lack of public confidence in the health system. Lack of capacity included limited numbers of healthcare facilities in general, but especially in rural areas, few healthcare facilities providing screening services, limited staff, brief and rushed consultations, and shortage of equipment and materials which often led to women being referred for screening far from where they live resulting in costly, and lengthy screening and diagnostic pathways [17–19, 24, 25, 30, 35, 37, 39, 41, 44, 47, 48, 52–55, 58, 61, 74, 82, 85, 87, 89].

Capacity barriers also included reports of poor knowledge of cervical cancer among healthcare workers, poor technical skill to perform screening procedures, limited supervision leaving staff uncertain about technique, and limited specialized experts such as gynaecologists for guidance and management of some patients [15, 25, 54, 77]. In Kenya and Ethiopia, clinic operating times and unavailability of services on weekends also limited screening uptake [13, 51]. In studies conducted in Uganda and South Africa, women reported that lack of privacy in healthcare facilities was a barrier to screening [29, 48], while in Malawi, Munthali et al., identified a lack of space for screening services in healthcare facilities as a barrier [47]. Lack of confidence in the health system was reported in Nigeria and Uganda [40, 54].

Eleven studies, seven in Africa (*n* = 7), Asia (*n* = 3) and South America (*n* = 1) found that poor, negative and discriminatory attitudes of healthcare workers towards women discouraged women from screening [16, 25, 49–53, 59, 61, 65, 80]. A study conducted in Nigeria, reported that discrimination toward Muslim women hindered access to healthcare facilities and screening [40]. Two studies also found that communication and language barriers between women and healthcare workers left women with unanswered questions and limited screening uptake [15, 80].

Long wait times in healthcare facilities were a barrier to screening in South Africa, Uganda, Kenya and China [16, 26, 43, 48, 50, 53, 61]. This may partly also explain why women reported competing priorities for their time (work and family responsibilities) when they considered attending screening services.

Several studies reported on policy and guideline implementation barriers. Studies in Uganda, Indonesia, Brazil, and China found poor organisation of the services with limited information available about screening services leaving women without information about screening sites, and procedures for booking screening appointments [48, 57, 61, 83]. In Bolivia, healthcare workers reported that lack of dissemination of screening guidelines, and lack of educational campaigns and infrastructure for screening limited screening uptake [82]. In Oceania, screening guidelines were not implemented while Bulgaria had no screening policy [85, 86]. In Argentina and China, the screening policy excluded unmarried women from free screening (in China), thus limiting screening for some women since out-of-pocket screening costs were frequently identified as a barrier to uptake [61, 84]. Healthcare workers also often failed to promote, recommend or offer screening and related cervical cancer information during other consultations [18, 38, 43, 62, 71, 74].

### Structural barriers

Structural barriers were mainly related to geographic distance to screening facilities, associated travel costs, poor transport systems, and screening costs where screening was not a free service in the absence of health insurance. Screening costs were a barrier in all continents, with travels costs a barrier in Africa, Asia and South America [12, 15, 18, 23, 25, 31, 33–35, 41, 43, 44, 46–48, 51, 59, 60, 79, 84, 86]. Long waiting times were also associated with additional costs for meals, and this increased overall screening costs [55]. Women in rural areas were disproportionately affected by distance, and travel costs [10, 16, 44, 76, 78]. In South Africa, Uganda and Nigeria, additional barriers were crime (which hindered free and safe travel), poor road networks and unreliable and inconvenient transport schedules to screening facilities [10, 44, 54]. One study reported lack of infrastructure for women with disabilities [11]. Other structural issues included low levels of education and low socioeconomic status [27, 32, 34], common among women living in conditions of poverty or limited resources.

### Underlying reasons for barriers to screening uptake

Based on the descriptive analysis of the main findings of the studies included in this review, we identified four underlying reasons for barriers to cervical cancer screening uptake that should be addressed when considering interventions and policies for remedial action. Firstly, poor or ineffective messaging about cervical cancer, screening and prevention evidenced by limited information and education about cervical cancer and screening as a preventive strategy and misconceptions about the cause of cervical cancer, and the screening process, is a key underlying reason for poor screening uptake. Many women are not aware of screening and its value, and there are many misconceptions about screening in many communities. Secondly, health systems are poorly resourced to provide screening, lack clear policies on cervical cancer and screening, or poorly implement any existing policies [48, 57, 60, 61, 82, 85, 86]. Thirdly, there is limited access to health care services more generally, because of lack of universal health coverage and affordability, a common feature in many LMICs and a notable barrier to screening uptake [15, 18, 25, 28, 31, 34, 35, 41, 46, 48, 51, 59, 61, 85]. Women often must travel to facilities far from where they live for screening services, indicating limited access in many geographic areas which is worsened by transport and other additional costs [15, 18, 22, 23].

Finally, gender norms that deprioritize the health needs of women both at institutional, community and household levels also underly poor screening uptake [13, 20, 22, 25, 30, 33–35, 41, 47, 48, 51, 60, 64, 65, 74]. Patriarchal norms which value the needs of men and boys over women and girls are often upheld in institutions and communities, which shapes political will and decision-making regarding investment in women’s health and creates inequitable health and access to care for women [90, 91]. In many studies, women reported a lack of partner approval, permission, or support, as well as religious, cultural, or traditional prohibitions as a barrier to uptake, indicating the breadth and depth of the impact of gender norms.

## Discussion

This review provides a broad overview of the barriers to uptake of cervical cancer screening in LMICs. The barriers were generally the same across countries and continents and different study designs, and are attributable to interacting individual, social, cultural, health system and structural factors.

At the individual level, lack of knowledge and information about cervical cancer, the screening process, and its value, were frequently reported. This suggests that failure to address the knowledge and information gaps, will likely continue to limit uptake even in the absence of other barriers. The literature also reports poor uptake among well-informed women, who reported other barriers rooted in societal religious, cultural health system and structural barriers [92–97]. Another common individual level barrier was fear which encompassed a wide range of issues. Limited information about the screening process (how it is done and by whom), may result in fear of what to expect. In Switzerland, women preferred to screen themselves using the self-HPV test kit since it reduced discomfort, embarrassment and maintained privacy compared to the traditional pap smear test [97]. Appropriate and careful introduction and scale up of such self-testing could expand screening in LMICs. Fear of the screening outcome could indicate anxiety around stigmatization, related to discrimination of women with cervical cancer. In a Ugandan study, cervical cancer patients were abandoned by their families, while in a Zambian study, cervical cancer was associated with shame [82, 98]. Stigma has also been reported in high income countries. Muslim women in London were hesitant to screen due to embarrassment and fear because they were unmarried and did not want to send implicit messages about being sexually active [99]. Another study also in the United Kingdom found that cervical cancer screening was stigmatized because of its association with HPV, and the perception that it shows failure of women’s responsibility for their health [100]. This emphasizes the urgent need for strengthened information dissemination, attention to gender-related discrimination, and dispelling of myths, about cervical cancer.

Cultural/traditional, religious, and social barriers were identified across many studies in all continents, but mainly in Africa and Asia. Lack of spousal and or family support were key barriers, and these may be driven by misconceptions about cervical cancer and traditional, cultural, or religious beliefs about pelvic examination and cancers, and this has also been reported in high income countries [101, 102]. Overlapping with cultural/traditional and religious barriers were other social factors including misconceptions and stigmatization of screening and cervical cancer, largely shaped by gender norms [14, 26, 33, 48, 58]. The impact of gender norms and inequality were common barriers. When men hold decision-making power, women and girls can have limited access to the social, economic and health resources necessary for their well-being [91]. At the household level, men often shape the logistical, educational, and psychosocial factors that directly affect women’s ability to access cervical cancer services. Women who are emotionally and financially supported by their families and partners are more likely to get screened. Conversely, family and partners can play a key role in stigmatizing, isolating, and prohibiting women from accessing screening.

Well-functioning health systems with accessible services are critical for successful and effective health programmes. We found significant gaps in cervical cancer screening services in the health systems of LMICs ranging from a lack of high-level elements such as policies and guidelines, poor referral systems, limited points of service, inadequate resources (human and equipment/materials), to local level factors including poor attitudes of healthcare workers. Poor attitudes and discrimination by healthcare workers while inexcusable may be fuelled by staff overload and challenging and constrained conditions [47, 103, 104], areas in need of urgent attention of policy makers and implementers.

Access to screening services was also hindered by geography and cost. Travel costs are significant for women with limited financial means. Women with low levels of education – who often have limited financial means – were less likely to be screened, hence, investing in women’s education in combination with other equity-promoting interventions is likely to improve uptake, given the known benefits of education.

### Strengths and limitations

This review includes a wide range of studies (both qualitative, quantitative, and mixed method study designs) and grey literature published over the period 2010 and 2020, enabling an extensive investigation of barriers to cervical cancer screening in LMICs. However, a potential limitation is that studies may have been overlooked due to the search terms used. For example, if studies used terms other than “Vaginal Smears”, “Papanicolaou”, “pap smear”, “pap stain”, “pap test” or “vaginal smear” to describe this specific screening test, they may not be included in the search results. We also included studies where barriers to cervical screening uptake was not a primary objective, and this may limit generalizability of some findings. However, the common barriers were corroborated by many different studies, looking at multiple level barriers to screening in LMICs.

### Recommendations

To increase screening uptake and support the elimination of cervical cancer as a public health problem in LMICs, there is a need for implementation of clear cervical cancer policies and guidelines with the prerequisite structures and resources required across diverse health settings. Countries should review their cervical cancer policies and related programs, and fully implement screening guidelines which prioritize structured screening, rather than rely on opportunistic screening that is patient driven. Policies – both within and beyond the health sector – should also actively account for and work to eliminate stigma and all forms of disadvantage and discrimination that shape inequities in communities and within the healthcare system. There is also need for education, information dissemination, engagement, and advocacy about cervical cancer at the community and health facility level. This creation of knowledge and awareness amongst community members and providers around how to proactively reduce barriers to care is crucial for ensuring more women receive screening, and is central to addressing misconceptions, myths, and fears that are prevalent in many communities. Education and awareness initiatives should be grounded in accessible language, driven by local and community contexts and needs, and meaningfully engage diverse groups of women, men, boys and girls as well as multiple sector stakeholders (including a community health worker component focused on women’s health and counselling). Policies that support the sexual and reproductive health and rights of women and girls should be strengthened and expanded and account for inequities in access to care for diverse groups of women. This can include culturally appropriate interventions with a dedicated focus on promoting women’s health, taking into account the social and financial needs of communities. Further priorities at the health facility level includes adequately addressing issues around staff-patient ratio, staff capacities and competencies, organization and integration of facility services, and health promotion efforts aimed at attracting community members for screening. To engage women and communities effectively and consistently, outreach efforts should be conducted in a manner that recognises the different contexts with regards to physical access, affordability, culture, tradition, and competence of health providers to provide high quality and friendly services. Community and religious leaders, non-governmental organizations (NGOs), women who have been screened, and other stakeholders need to reinforce and advocate the message that screening saves lives. This would be an important step in combatting the stigma related to cervical cancer screening.

Future research should focus on generating robust data on which groups are under-screened and why. This must account for the differential experiences of women across diverse categories (e.g., age, socioeconomic status, geography, disability, etc.) and look at the multiple level barriers that converge to create or reinforce barriers to health and screening. 

This review highlights some of the key issues highlighted in the literature to date, but there remains a dearth of information as to the multi-level barriers to screening that women face across axes of inequity, including gender, age, income, migrant status, ability, etc.

Finally, the introduction of more modern screening approaches in LMICs should also be supported. It is better information, better resources, and input from women themselves, that can ground how barriers are addressed and how access is improved moving forward.

## Conclusion

This review identified a wide range of barriers to cervical cancer screening in LMICs. Urgent implementation of clear policies and programs, supported by health system capacity to implement them is required to address these barriers. The policies should support the promotion of women and girls’ health and rights, and gender equality. In addition, community-wide information dissemination, engagement and advocacy, and targeted further research on barriers to care across diverse groups and contexts are needed to effectively address the inequitable burden of cervical cancer in LMICs. It is only in reducing the barriers to cervical cancer screening that so many women continue to face, that the aims of the WHO’s global strategy to eliminate cervical cancer as a public health problem will be fulfilled.

## Supplementary Information

**Additional file 1: Appendix 1.** Quality assessment of studies.

## Data Availability

The articles reviewed and/or analysed during the current study are available from the corresponding author on reasonable request.

## References

[1] IARC and WHO. GLOBOCAN 2018: Estimated cancer incidence, mortality and prevalence worldwide in 2018. Cervical cancer Fact Sheet. Available at: https://gco.iarc.fr/today/data/factsheets/cancers/23-Cervix-Uteri-fact-sheet.pdf.

[2] World health Organization (2020). Global strategy to accelerate the elimination of cervical cancer as a public health problem.

[3] Arbyn M, Weiderpass E, Bruni L, de Sanjosé S, Saraiya M, Ferlay J, Bray F (2020). Estimates of incidence and mortality of Cervical cancer in 2018: a worldwide analysis. Lancet Glob Health.

[4] Gakidou E, Nordhagen S, Obermeyer Z (2008). Coverage of cervical cancer screening in 57 countries: low average levels and large inequalities. PLoS Med.

[5] Bronfenbrenner U, Friedman SL, Wachs TD (1999). Measuring environment across the life span: Emerging methods and concepts.

[6] Bronfenbrenner U (1979). The ecology of human development: Experiments by nature and design.

[7] Hannes K, Macaitis K (2012). A move to more systematic and transparent approaches in qualitative evidence synthesis: update on a review of published papers. Qual Res.

[8] Critical Appraisal Skills Programme (CASP) (2015). Critical Appraisal Skills Programme (CASP).

[9] Calys-Tagoe BN, Aheto JM, Mensah G, Biritwum RB, Yawson AE (2020). Cervical cancer screening practices among women in Ghana: evidence from wave 2 of the WHO study on global AGEing and adult health. BMC Womens Health.

[10] Stewart K, Li M, Xia Z (2020). Modeling spatial access to Cervical cancer screening services in Ondo State, Nigeria. Int J Health Geogr.

[11] Park SJ, Park WS (2010). Identifying barriers to Papanicolaou smear screening in Korean women: Korean National Health and Nutrition Examination Survey 2005. J Gynecol Oncol.

[12] Gottschlich A, Rivera-Andrade A, Bevilacqua K (2020). Using self-collection HPV testing to increase engagement in Cervical cancer screening programs in rural Guatemala: a longitudinal analysis. BMC Public Health.

[13] Getahun T, Kaba M, Derseh BT. Intention to screen for cervical cancer in debre berhan town, amhara regional state, ethiopia: application of theory of planned behavior. J Cancer Epidemiol. 2020;2020:8. 10.1155/2020/3024578. 10.1155/2020/3024578 PMC7106926 32256590

[14] Megersa BS, Bussmann H, Bärnighausen T, Muche AA, Alemu K, Deckert A (2020). Community Cervical cancer screening: Barriers to successful home-based HPV self-sampling in Dabat district, North Gondar, Ethiopia. A qualitative study. PLoS ONE.

[15] Ampofo AG, Adumatta AD, Owusu E, Awuviry-Newton K (2020). A cross-sectional study of barriers to Cervical cancer screening uptake in Ghana: An application of the health belief model. PLoS One.

[16] Harries J, Scott SE, Walter FM (2020). Women’s appraisal, interpretation and help-seeking for possible symptoms of breast and Cervical cancer in South Africa: a qualitative study. BMC Womens Health.

[17] Nyamambi E, Murendo C, Sibanda N, Mazinyane N (2020). Knowledge, attitudes and barriers of Cervical cancer screening among women in Chegutu rural district of Zimbabwe. Cogent Soc Sci.

[18] Getachew S, Getachew E, Gizaw M, Ayele W, Addissie A, Kantelhardt EJ (2019). Cervical cancer screening knowledge and barriers among women in Addis Ababa, Ethiopia. PLoS One.

[19] Nigussie T, Admassu B, Nigussie A (2019). Cervical cancer screening service utilization and associated factors among age-eligible women in Jimma town using health belief model, Southwest Ethiopia. BMC Women’s Health.

[20] Solomon K, Tamire M, Kaba M (2019). Predictors of Cervical cancer screening practice among HIV positive women attending adult anti-retroviral treatment clinics in Bishoftu town, Ethiopia: the application of a health belief model. BMC Cancer.

[21] Williams MS, Kenu E, Adanu A, Yalley RA, Lawoe NK, Dotse AS, Adu RF, Fontaine K (2019). Awareness and Beliefs About Cervical cancer, the HPV Vaccine, and Cervical cancer Screening Among Ghanaian Women with Diverse Education Levels. J Cancer Educ.

[22] Adewumi K, Oketch SY, Choi Y (2019). Female perspectives on male involvement in a human-papillomavirus-based Cervical cancer-screening program in western Kenya. BMC Womens Health.

[23] Oketch SY, Kwena Z, Choi Y (2019). Perspectives of women participating in a Cervical cancer screening campaign with community-based HPV self-sampling in rural western Kenya: a qualitative study. BMC Womens Health.

[24] Lieber M, Afzal O, Shaia K, Mandelberger A, Du Preez C, Beddoe AM (2019). Cervical cancer Screening in HIV-Positive Farmers in South Africa: Mixed-Method Assessment. Ann Glob Health.

[25] Shiferaw S, Addissie A, Gizaw M, Hirpa S, Ayele W, Getachew S, Kantelhardt EJ, Assefa M, Jemal A (2018). Knowledge about Cervical cancer and barriers toward Cervical cancer screening among HIV-positive women attending public health centers in Addis Ababa city, Ethiopia. Cancer Med.

[26] Kangmennaang J, Onyango EO, Luginaah I, Elliott SJ (2018). The next Sub-Saharan African epidemic? A case study of the determinants of Cervical cancer knowledge and screening in Kenya. Soc Sci Med.

[27] Ng'ang'a A, Nyangasi M, Nkonge NG, Gathitu E, Kibachio J, Gichangi P, Wamai RG, Kyobutungi C (2018). Predictors of Cervical cancer screening among Kenyan women: results of a nested case-control study in a nationally representative survey. BMC Public Health.

[28] Maree JE, Kampinda-Banda M. Knowledge and practices of cervical cancer and its prevention among Malawian women. J Cancer Educ. 2020;35(1):86-92. 10.1007/s13187-018-1443-4 30415315

[29] Keneema M. Factors Affecting Uptake Of Cervical Cancer Screening Services Among Women Aged 25-49 Attending Antenatal Clinic At Rukunyu Health Center Iv, Kamwenge District (Doctoral dissertation, International Health Sciences University); 2018.

[30] Vhuromu EN, T Goon D, Maputle MS, Lebese RT, Okafor BU. Utilization of cervical cancer screening services among women in Vhembe District, South Africa: a cross-sectional study. Open Public Health J. 2018;11(1):451-463.

[31] Kokuro M (2017). Factors affecting the utilisation of Cervical cancer screening among women attending health services in the kumasi metropolis of Ghana (Doctoral dissertation, Stellenbosch: Stellenbosch University).

[32] Bishwajit G, Kpoghomou MA (2017). Urban-rural differentials in the uptake of mammography and Cervical cancer screening in Kenya. J Cancer Policy.

[33] Lunsford NB, Ragan K, Smith JL, Saraiya M, Aketch M (2017). Environmental and Psychosocial Barriers to and Benefits of Cervical cancer Screening in Kenya. Oncologist..

[34] Tiruneh FN, Chuang KY, Ntenda P, Chuang YC (2017). Individual-level and community-level determinants of Cervical cancer screening among Kenyan women: a multilevel analysis of a Nationwide survey. BMC Womens Health.

[35] Filade TE, Dareng EO, Olawande T, Fagbohun TA, Adebayo AO, Adebamowo CA (2017). Attitude to Human Papillomavirus Deoxyribonucleic Acid-Based Cervical cancer Screening in Antenatal Care in Nigeria: A Qualitative Study. Front Public Health.

[36] Momberg M, Botha MH, Van der Merwe FH, Moodley J (2017). Women’s experiences with Cervical cancer screening in a colposcopy referral clinic in Cape Town, South Africa: a qualitative analysis. BMJ Open.

[37] Malambo N, Erikson S (2018). ‘Worse than HIV’: The logics of cancer screening avoidance in Swaziland. Glob Public Health.

[38] Mitchell SM, Pedersen HN, Eng Stime E, Sekikubo M, Moses E, Mwesigwa D, Biryabarema C, Christilaw J, Byamugisha JK, Money DM, Ogilvie GS (2017). Self-collection-based HPV testing for Cervical cancer screening among women living with HIV in Uganda: a descriptive analysis of knowledge, intentions to screen and factors associated with HPV positivity. BMC Womens Health.

[39] Koneru A, Jolly PE, Blakemore S, McCree R, Lisovicz NF, Aris EA (2017). Acceptance of peer navigators to reduce barriers to Cervical cancer screening and treatment among women with HIV infection in Tanzania. Int J Gynaecol Obstet.

[40] Modibbo FI, Dareng E, Bamisaye P, Jedy-Agba E, Adewole A, Oyeneyin L, Olaniyan O, Adebamowo C (2016). Qualitative study of barriers to Cervical cancer screening among Nigerian women. BMJ Open.

[41] Hweissa NA, Lim JNW, Su TT (2016). Health-care providers’ perceptions, attitudes towards and recommendation practice of Cervical cancer screening. Eur J Cancer Care (Engl).

[42] Adepoju EG, Ilori T, Olowookere SA, Idowu A. Targeting women with free cervical cancer screening: challenges and lessons learnt from Osun state, southwest Nigeria. Pan Afr Med J. 2016;24. 10.11604/pamj.2016.24.319.9300 PMC5267846 28154674

[43] Ndejjo R, Mukama T, Musabyimana A, Musoke D (2016). Uptake of Cervical cancer Screening and Associated Factors among Women in Rural Uganda: A Cross Sectional Study. PLoS One.

[44] Hasahya OT, Berggren V, Sematimba D, Nabirye RC, Kumakech E (2016). Beliefs, perceptions and health-seeking behaviours in relation to Cervical cancer: a qualitative study among women in Uganda following completion of an HPV vaccination campaign. Glob Health Action.

[45] Ghidei L. Knowledge and Perception of Cervical Cancer and Screening Programs of Women Seeking Care at Monduli Hospital in Tanzania and St. Paul Hospital in Addis Ababa, Ethiopia (Doctoral dissertation); 2015.

[46] Compaore S, Ouedraogo CMR, Koanda S, Haynatzki G, Chamberlain RM, Soliman AS. Barriers to Cervical cancer Screening in Burkina Faso: Needs for Patient and Professional Education. J Cancer Educ. 2015:760–6. 10.1007/s13187-015-0898-9 PMID: 26336956; PMCID: PMC4779069. 10.1007/s13187-015-0898-9 PMC4779069 26336956

[47] Munthali AC, Ngwira BM, Taulo F (2015). Exploring barriers to the delivery of Cervical cancer screening and early treatment services in Malawi: some views from service providers. Patient Preference Adherence.

[48] Learmonth D, Hakala S, Keller M (2015). “I can't carry on like this”: barriers to exiting the street-based sex trade in South Africa. Health Psychol Behav Med.

[49] Ebu NI, Mupepi SC, Siakwa MP, Sampselle CM (2014). Knowledge, practice, and barriers toward Cervical cancer screening in Elmina, Southern Ghana. Int J Womens Health.

[50] Omondi Aduda DS, Mkhize N. Ethical issues evolving from patients’ perspectives on compulsory screening for syphilis and voluntary screening for cervical cancer in Kenya. BMC medical ethics. 2014;15(1):1-2. 10.1186/1472-6939-15-27 PMC3973750 24678613

[51] Kibicho JW (2014). Factors influencing utilization of Cervical cancer screening services in Embu hospital, Embu County, Kenya Master of Arts Degree in Project Planning and Management, University of Nairobi.

[52] Abdulkadir IR (2013). Level of knowledge toward human papillomavirus/Cervical cancer & practice of Papanicolaou test screening among female Addis Ababa university students in Ethiopia.

[53] Atuhaire L. Barriers and facilitators to uptake if cervical cancer screening among women accessing maternal and child health services in Kampala, Uganda; 2013. Available from: http://hdl.handle.net/11394/3924.

[54] Mwaka AD, Wabinga HR, Mayanja-Kizza H (2013). Mind the gaps: a qualitative study of perceptions of healthcare professionals on challenges and proposed remedies for Cervical cancer help-seeking in post conflict northern Uganda. BMC Fam Pract.

[55] Paul P, Winkler JL, Bartolini RM, Penny ME, Huong TT, Nga LT, Jeronimo J (2013). Screen-and-treat approach to Cervical cancer prevention using visual inspection with acetic acid and cryotherapy: experiences, perceptions, and beliefs from demonstration projects in Peru, Uganda, and Vietnam. Oncologist.

[56] Ngugi CW, Boga H, Muigai AW, Wanzala P, Mbithi JN (2012). Factors affecting uptake of Cervical cancer early detection measures among women in Thika, Kenya. Health Care Women Int.

[57] Hyacinth HI, Adekeye OA, Ibeh JN, Osoba T (2012). Cervical cancer and pap smear awareness and utilization of pap smear test among Federal civil servants in North Central Nigeria. PLoS One.

[58] Mupepi SC, Sampselle CM, Johnson TR (2011). Knowledge, attitudes, and demographic factors influencing Cervical cancer screening behavior of Zimbabwean women. J Women’s Health (Larchmt).

[59] Andersen JG, Shrestha AD, Gyawali B, Neupane D, Kallestrup P. Barriers and facilitators to Cervical cancer screening uptake among women in Nepal – a qualitative study. Women Health. 2020. 10.1080/03630242.2020.1781742. 10.1080/03630242.2020.1781742 32643576

[60] Spagnoletti B, Bennett LR, Wahdi AE, Wilopo SA, Keenan CA (2019). A Qualitative Study of Parental Knowledge and Perceptions of Human Papillomavirus and Cervical cancer Prevention in Rural Central Java, Indonesia: Understanding Community Readiness for Prevention Interventions. Asian Pac J Cancer Prev.

[61] Gu C, Chan CW, Chow KM, Yang S, Luo Y, Cheng H, Wang H (2018). Understanding the cervical screening behaviour of Chinese women: The role of health care system and health professions. Appl Nurs Res.

[62] Ashtarian H, Mirzabeigi E, Mahmoodi E, Khezeli M (2017). Knowledge about Cervical cancer and Pap Smear and the Factors Influencing the Pap test Screening among Women. Int J Community Based Nurs Midwifery.

[63] Östh J (2015). Knowledge of Human Papilloma Virus, Cervical cancer and Cytological Screening and Attitudes towards and Practices of Screening among Undergraduate students at Rajarata University, Sri Lanka: A cross-sectional study.

[64] Jia Y, Li S, Yang R, Zhou H, Xiang Q, Hu T, Zhang Q, Chen Z, Ma D, Feng L (2013). Knowledge about Cervical cancer and barriers of screening program among women in Wufeng County, a high-incidence region of Cervical cancer in China. PLoS One.

[65] Baskaran P, Subramanian P, Rahman RA, Ping WL, Mohd Taib NA, Rosli R (2013). Perceived susceptibility, and Cervical cancer screening benefits and barriers in Malaysian women visiting outpatient clinics. Asian Pac J Cancer Prev.

[66] Gan DEH, Dahlui M. Cervical screening uptake and its predictors among rural women in Malaysia. Singapore Med J. 2013;54(3):163-168. 10.11622/smedj.2013047 23546031

[67] Demirtas B, Acikgoz I (2013). Promoting attendance at Cervical cancer screening: understanding the relationship with Turkish womens’ health beliefs. Asian Pac J Cancer Prev.

[68] Guvenc G, Akyuz A, Yenen MC. Effectiveness of nursing interventions to increase pap smear test screening. Res Nurs Health. 2013;36(2):146-57. 10.1002/nur.21526 23335354

[69] Reis N, Bebis H, Kose S, Sis A, Engin R, Yavan T (2012). Knowledge, behavior and beliefs related to Cervical cancer and screening among Turkish women. Asian Pac J Cancer Prev.

[70] Gu C, Chan CW, Twinn S, Choi KC. The influence of knowledge and perception of the risk of cervical cancer on screening behavior in mainland Chinese women. Psycho-Oncology. 2012;21(12):1299-308. 10.1002/pon.2037 23208838

[71] Abdullah F, Aziz NA, Su TT (2011). Factors related to poor practice of Pap smear screening among secondary school teachers in Malaysia. Asian Pac J Cancer Prev.

[72] Gu C, Chan CW, Twinn S (2010). How sexual history and knowledge of Cervical cancer and screening influence Chinese women's screening behavior in mainland China. Cancer Nurs.

[73] Abdullah F, Su TT (2010). Enhancement of the Cervical cancer screening program in Malaysia: a qualitative study. Asian Pac J Cancer Prev.

[74] Al-Naggar RA, Low WY, Isa ZM (2010). Knowledge and barriers towards Cervical cancer screening among young women in Malaysia. Asian Pac J Cancer Prev.

[75] Bien-Aimé, Danta Dona Ruthnie. Understanding the Barriers and Facilitators to Cervical Cancer Screening Among Women in Gonaives, Haiti: An Explanatory Sequential Mixed-Methods Study. Master’s thesis, Harvard Medical School; 2020. Available from: https://nrs.harvard.edu/URN-3:HUL.INSTREPOS:37365189.

[76] Lyons KD, Kennedy LS, Larochelle EPM, Tsongalis GJ, Reyes HS, Zuniga-Moya JC, Chamberlin MD, Bruce ML, Bejarno S (2020). Feasibility of Brigade-Style, Multiphasic Cancer Screening in Rural Honduras. JCO Glob Oncol.

[77] Chary AN, Rohloff PJ (2014). Major challenges to scale up of visual inspection based Cervical cancer prevention programs: the experience of Guatemalan NGOs. Global Health Sci Pract.

[78] Barrett BW, Paz-Soldan VA, Mendoza-Cervantes D, Sánchez GM, Córdova López JJ, Gravitt PE, Rositch AF, Proyecto Precancer Study Group (2020). Understanding Geospatial Factors Associated with Cervical cancer Screening Uptake in Amazonian Peruvian Women. JCO Global Oncol.

[79] Collins JH, Bowie D, Shannon G. A descriptive analysis of health practices, barriers to healthcare and the unmet need for Cervical cancer screening in the Lower Napo River region of the Peruvian Amazon. Women’s Health. 2019. 10.1177/1745506519890969. 10.1177/1745506519890969 PMC6918491 31840562

[80] Nugus P, Désalliers J, Morales J, Graves L, Evans A, Macaulay AC (2018). Localizing Global Medicine: Challenges and Opportunities in Cervical Screening in an Indigenous Community in Ecuador. Qual Health Res.

[81] Albuquerque CL, Costa Mda P, Nunes FM, Freitas RW, Azevedo PR, Fernandes JV, Rego JV, Barreto HM (2014). Knowledge, attitudes and practices regarding the Pap test among women in North-eastern Brazil. Sao Paulo Med J.

[82] Stormo AR, Altamirano V, Pérez-Castells M, Espey D, Padilla H, Panameño K, Soria M, Santos C, Saraiya M, Luciani S (2012). Bolivian health providers’ attitudes toward alternative technologies for Cervical cancer prevention: a focus on visual inspection with acetic acid and cryotherapy. J Women’s Health.

[83] Paz-Soldán V, Bayer A, Nussbaum L, Cabrera L (2012). Structural barriers to screening for and treatment of Cervical cancer in Peru. Reprod Health Matters.

[84] Paolino M, Arrossi S (2011). Women’s knowledge about Cervical cancer, Pap smear and human papillomavirus and its relation to screening in Argentina. Women Health.

[85] Townsend JS, Stormo AR, Roland KB, Buenconsejo-Lum L, White S, Saraiya M (2014). Current Cervical cancer screening knowledge, awareness, and practices among U.S. affiliated pacific island providers: opportunities and challenges. Oncologist.

[86] Valerianova Z, Panayotova Y, Amati C, Baili P (2010). Cervical cancer Screening in Bulgaria - past and present Experience. Tumori..

[87] Rada C, Hudiţa D, Manolescu S, Prejbeanu I, Zugravu CA (2010). Attitudinal and behavioral patterns, socio-demographical characteristics of risk for cervical cancer. Gineco.ro..

[88] Lisy K, Porritt K (2016). Narrative synthesis: considerations and challenges. JBI Evid Implement.

[89] Maree JE, Wright SC (2011). Cervical cancer: does our message promote screening? A pilot study in a South African context. Eur J Oncol Nurs.

[90] Sen G, Östlin P (2008). Unequal, Unfair, Ineffective and Inefficient; Gender Inequity in Health: Why it exists and how we can change it. WHO Commission on Social Determinants of Health.

[91] United nations 2016 Report of the Working Group on the issue of discrimination against women in law and in practice. https://documents-dds-ny.un.org/doc/UNDOC/GEN/G16/072/19/PDF/G1607219.pdf?OpenElement.

[92] Chorley AJ, Marlow LA, Forster AS, Haddrell JB, Waller J (2017). Experiences of cervical screening and barriers to participation in the context of an organised programme: a systematic review and thematic synthesis. Psycho-Oncology..

[93] Akinlotan M, Bolin JN, Helduser J, Ojinnaka C, Lichorad A, McClellan D (2017). Cervical cancer screening barriers and risk factor knowledge among uninsured women. J Community Health.

[94] Selmouni F, Zidouh A, Alvarez-Plaza C, El Rhazi K (2015). Perception and satisfaction of cervical cancer screening by Visual Inspection with Acetic acid (VIA) at Meknes-Tafilalet Region, Morocco: a population-based cross-sectional study. BMC Womens Health.

[95] Bates CK, Carroll N, Potter J (2011). The challenging pelvic examination. J Gen Intern Med.

[96] Quincy BL, Turbow DJ, Dabinett LN (2012). Acceptability of self-collected human papillomavirus specimens as a primary screen for cervical cancer. J Obstet Gynaecol.

[97] Fargnoli V, Petignat P, Burton-Jeangros C (2015). To what extent will women accept HPV self-sampling for cervical cancer screening? A qualitative study conducted in Switzerland. Int J Women's Health.

[98] Rahman R, Clark MD, Collins Z, Traore F, Dioukhane EM, Thiam H, Dykens JA (2019). Cervical cancer screening decentralized policy adaptation: an African rural-context-specific systematic literature review. Glob Health Action.

[99] Szarewski A, Cadman L, Ashdown-Barr L, Waller J (2009). Exploring the acceptability of two self-sampling devices for human papillomavirus testing in the cervical screening context: a qualitative study of Muslim women in London. J Med Screen.

[100] Vrinten C, Wardle J, Marlow LA (2016). Cancer fear and fatalism among ethnic minority women in the United Kingdom. Br J Cancer.

[101] Padela AI, Peek M, Johnson-Agbakwu CE, Hosseinian Z, Curlin F (2014). Associations between religion-related factors and cervical cancer screening among Muslims in greater Chicago. J Lower Genital Tract Disease.

[102] Salman KF (2012). Health beliefs and practices related to cancer screening among Arab Muslim women in an urban community. Health Care For Women International.

[103] Dykens JA, Smith JS, Demment M, Tina EM, Karen S, Irwin T (2020). Evaluating the implementation of Cervical cancer screening programs in low - resource settings globally: a systematized review. Cancer Causes Control.

[104] Pierz AJ, Randall TC, Castle PE, Adedimeji A, Ingabire C, Kubwimana G (2020). A scoping review: Facilitators and barriers of Cervical cancer screening and early diagnosis of breast cancer in Sub-Saharan African health settings. Gynecol Oncol Reports.

