# Supplementary material for: Barriers to uptake of cervical cancer screening services in low-and-middle-income countries: a systematic review
Source: BMC Womens Health. 2022 Dec 2;22:486. doi: 10.1186/s12905-022-02043-y (PMC9716693; doi:10.1186/s12905-022-02043-y)
Supplement: Supplementary file 1 — Additional file 1: Appendix 1. Quality assessment of studies. [file 12905_2022_2043_MOESM1_ESM.docx]

**Web appendix 1: Quality assessment of studies**

The qualitative checklist of the Critical Appraisal Skill Program (CASP) [6] tool was used to assess qualitative studies. Ten criteria: clear study objectives, appropriate methodology, appropriate study design, recruitment strategy, data collection, consideration of the relationship between researchers and participants, ethical issues, rigorous analysis, clear findings and value of the research were assessed, with each criterion scored from 1 to 3 based on the researcher’s subjective judgment. The scores were summed for a total score and the overall quality of each study was ranked as low (1-10), medium (11-20), or high (21-30). Quantitative studies were assessed using from The CASP modified tool on the following 9 qualities: clear study objectives, appropriate methodology, representative sample and power, response rate and validation of the instrument, reliability of the results, appropriate tables and graphs, appropriate statistical methods, important variables considered, and value of the research. Each criterion was also scored from 1 to 3 based on the researcher’s subjective judgment, for a total score, and these were ranked as low (1-9), medium (10-19), or high (20-30).
